# Factors influencing scavenger guilds and scavenging efficiency in Southwestern Montana

**DOI:** 10.1038/s41598-021-83426-3

**Published:** 2021-02-19

**Authors:** Morgan A. Walker, Maria Uribasterra, Valpa Asher, Wayne M. Getz, Sadie J. Ryan, José Miguel Ponciano, Jason K. Blackburn

**Affiliations:** 1 Spatial Epidemiology & Ecology Research Laboratory, Department of Geography, University of Florida, Gainesville, FL USA; 2 Emerging Pathogens Institute, University of Florida, Gainesville, FL USA; 3 Turner Enterprises Inc., 1123 Research Drive, Bozeman, MT USA; 4 Department of Environmental Sciences, Policy, and Management, University of California, Berkeley, 130 Mulford Hall, Berkeley, CA USA; 5 School of Mathematical Sciences, University of KwaZulu-Natal, Durban, South Africa; 6 Quantitative Disease Ecology and Conservation Laboratory, Department of Geography, University of Florida, Gainesville, FL USA; 7 College of Agriculture, Engineering, and Science, University of KwaZulu-Natal, Durban, South Africa; 8 Department of Biology, University of Florida, Gainesville, FL USA

**Keywords:** Ecology, Behavioural ecology

## Abstract

Scavenging of carrion shapes ecological landscapes by influencing scavenger population demography, increasing inter- and intra-specific interactions, and generating ecosystem services such as nutrient cycling and disease moderation. Previous research found the cues promoting, or the constraints limiting, an individual’s propensity or ability to scavenge vary widely, depending on anthropogenic and environmental factors. Here we investigated differences in scavenging patterns in a complex scavenger guild in Southwestern Montana. We used camera traps established at 13 carcass sites to monitor carcass detection, visitation, and consumption times, during 2016–2018 and generalized linear models to explore the influence of carcass characteristics, habitat features, and seasonality, on carcass selection and scavenging efficiency. We found that scavenger species diversity was higher at higher elevations and in grassland habitats. Scavenging efficiency was influenced *inter alia* by seasonality, distance to water, and elevation. We found that most carcass consumption was via facultative scavengers (bears, wolves, magpies, *Corvus* spp.) rather than turkey vultures, the only obligate scavengers in the study area. However, growing populations of turkey vultures may lead to increased competition with facultative scavengers over carrion, and could have cascading effects on food webs in this ecosystem.

## Introduction

The role of carrion in community ecology has only recently gained recognition as a critical resource in terrestrial ecosystems^1–4^. Decreases in the availability of carrion have been shown to alter scavenger population demography and increase mortality^5–7^. In addition, scavenging shapes inter- and intra-specific interactions through competition^8,9^; and carrion has been shown to have positive and direct effects on the biomass and nutritional content of plants^10–13^. While recent studies have shed light on the ecological^4,14^ and economic^15^ importance of vertebrate scavenging, findings regarding the roles cues and constraints play in respectively promoting and limiting an individual’s propensity or ability to scavenge vary widely^8,16^ among ecosystems^17^. Additionally, studies on naturally occurring carcasses are rare^18–20^. The possible factors influencing carrion use include the characteristics of the carcass itself^16,21^ (species, spatio-temporal location, disease status or cause of death), the ecology of the scavenging animal (species, body size, sociality, inter- and intra-specific dynamics), and abiotic factors^18,19^ (season, weather, habitat). Scavenging of carrion is a fundamental process associated with several ecosystem services, including decomposition, nutrient cycling, and disease moderation^4,22,23^.

Organisms that rely solely on carrion for survival and reproduction are referred to as obligate scavengers, and the only terrestrial vertebrates categorized as such are vultures^24^. Conversely, facultative scavengers are species that scavenge when the opportunity arises, but are not solely dependent on carrion for survival; these generalists comprise a much more diverse set of species^24^. In fact, facultative scavenging is quite common, and nearly all carnivores will capitalize on carrion resources when given the opportunity^8,14,25,26^.

This study provides insight into the biotic and abiotic factors influencing the diversity of vertebrate scavengers and their behavior and scavenging efficiency in southwestern Montana, USA. This northern temperate ecosystem supports one of the few intact carnivore guilds in North America^27^ but is also a system shown to be highly vulnerable to global climate change^28–30^. In fact, climate change has already been linked to shorter periods of deep snow in winter in Yellowstone National Park, USA (~ 70 km from our study site), leading to a reduction in the number of ungulates dying from starvation and consequently the supply of carcasses to carrion-reliant species during this period^31^. The specific goals of our study were: (i) to evaluate the diversity of the facultative scavenger guild in an ecosystem with fluctuating carrion availability; and, (ii) to determine the influence of carcass characteristics, habitat features, and seasonality on carcass selection and scavenging efficiency.

## Results

**Figure 1:**
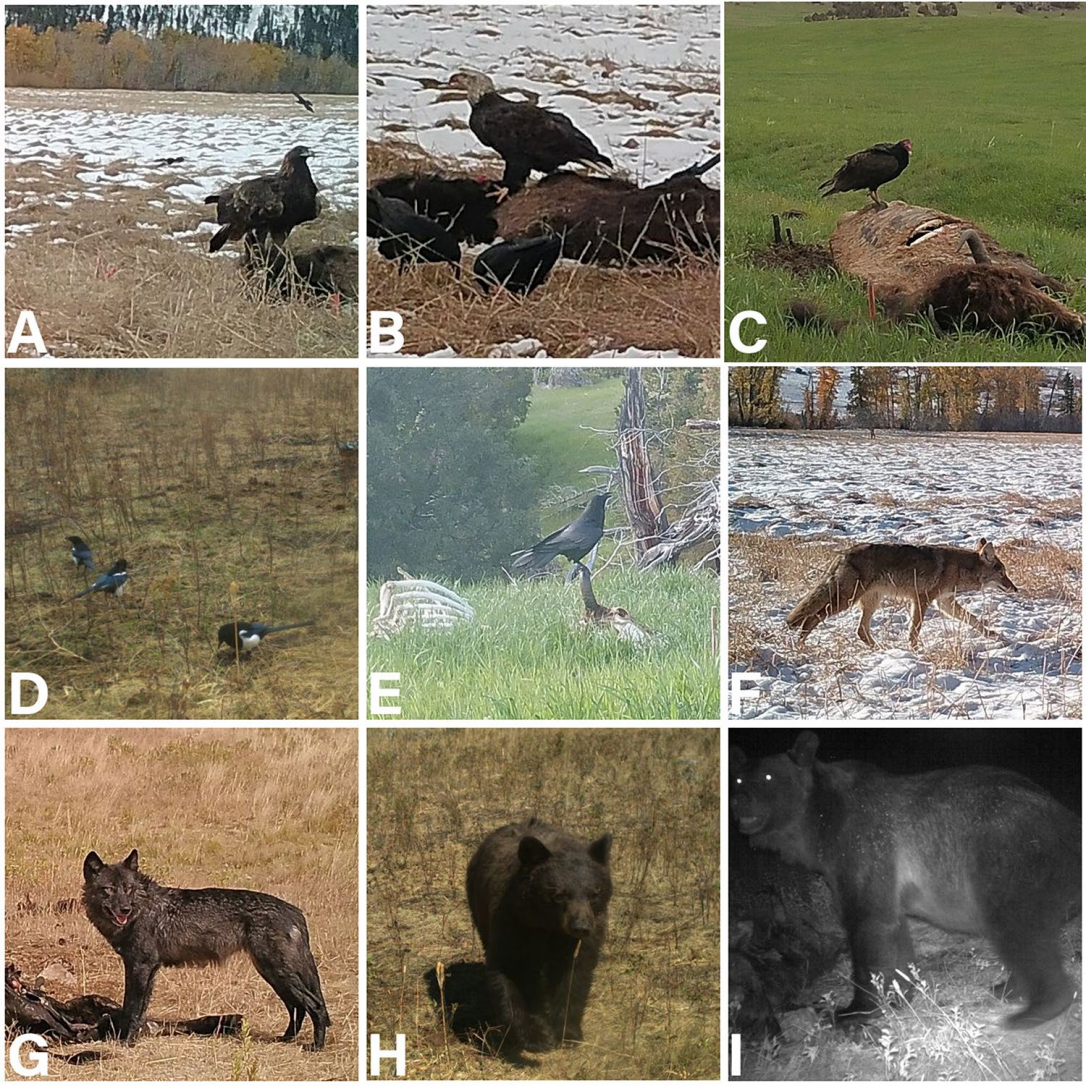
Images of scavenger species captured by motion-sensitive cameras deployed during a camera trap study of carcass use in southwestern Montana: golden eagles (**A**), bald eagles (**B**), turkey vultures (**C**), black-billed magpies (**D**), *Corvus* spp. (**E**), coyotes (**F**), wolves (**G**), black bears (**H**), brown bears (**I**). All images were captured by motion trigger.

From 2016 to 2018, we captured 725,421 camera trap photos. A total of 69,701 (9.61%) photos captured vertebrate scavengers. Of those, there were 21,257 (30.50%) photos of common ravens or American crows (‘*Corvus* spp.’; *Corvus corax* and *Corvus brachyrhynchos*), 16,025 (22.99%) of black-billed magpies (*Pica hudsonia*), 15,313 (21.97%) photos of coyotes (*Canis latrans*), 6,213 (8.91%) of golden eagles (*Aquila chrysaetos*), 3,219 (4.62%) of black bears (*Ursus americanus*), 3,163 (4.54%) of gray wolves (*Canis lupus*), 2,621 (3.76%) of bald eagles (*Haliaeetus leucocephalus*), 1,350 (1.94%) of brown bears (*Ursus arctos*), 454 (0.65%) of turkey vultures (*Cathartes aura*), and 86 (0.12%) of red fox (*Vulpes vulpes*) (Fig. 1). The only obligate scavengers^27^ photographed were turkey vultures; all other species listed are considered facultative scavengers.

The number of species visiting carcass sites varied from 2 to 9 species. The mean visiting species number across all 13 sites (i.e., ± SE) was 5.23 ± 0.68 species. The best generalized linear model (GLM) for the number of visiting species included only elevation as a predictor (AICc = 58.8, adj. *D*^2^ = 36%; Table1). Specifically, parameter estimates from this model indicated more scavenger species were present at carcass sites at higher elevations (*p* = 0.03; Table 2). There were two additional visiting species models with ΔAIC_c_ < 2; the second-ranked model included both elevation and carcass size (ΔAIC_c_ = 1.6, adj. *D*^2^ = 49%), while the third-ranked model included only carcass size (ΔAIC_c_ = 1.7, adj. *D*^2^ = 24%).

**Table 1:** AIC_c_-based model selection to assess the effects of carcass habitat, elevation, seasonality, carcass species, carcass body mass, and distance to both water and roads on scavenger guild numbers and scavenging efficiency at carcass sites in southwestern Montana.

Response variable	Model	AIC_c_	ΔAIC_c_	$\mathrm{adj} {D}^{2}$
Visiting species no	Elevation	58.8	0.0	36%
	Elevation + carcass size	60.4	1.6	49%
	Carcass size	60.5	1.7	24%
Consuming species no	Habitat	63.4	0.0	42%
	Season animal died	63.5	0.1	56%
	Elevation	63.8	0.3	29%
	Elevation + carcass size	64.4	1.0	38%
Detection time	Season animal died + distance to water + elevation + distance to road	107.5	0.0	87%
Consumption time	Season animal died + distance to water + carcass size	76.5	0.0	98%

Highest ranking models are shown as well as those models with a ΔAICc < 2. The quantity adj *D*^*2*^ is the percent deviance explained by each model.

**Table 2:** Top-ranked GLMs showing the relationship between visiting and consuming species numbers, scavenging efficiency (detection time and consumption time), and the following predictor variables: carcass size, elevation, season of animal death, distance to water, and distance to roads.

Response variable	Model	Parameter	Estimate	SE	*p*-value
Visiting species no	Elevation	Intercept	1.6152	0.1262	< 2e−16
		Elevation	0.2968	0.1346	0.0274
Consuming species no	Habitat	Intercept	1.0986	0.5774	0.0571
		Habitat (grassland)	0.6444	0.5986	0.2817
		Habitat (shrubland)	− 0.5108	0.6667	0.4435
Detection time	Season animal died + distance to water + elevation + distance to road	Intercept	5.0747	0.6270	5.78e−16
		Season animal died (spring)	− 6.3859	1.4308	8.08e−06
		Season animal died (summer)	2.6938	0.4967	5.86e−08
		Distance to water (meters)	− 1.8846	1.0398	2.06e−07
		Elevation (meters)	− 5.1266	0.6559	8.21e−07
		Distance to road (meters)	2.5036	0.3628	0.000135
Consumption time	Season animal died + distance to water + carcass size	Intercept	2.4228	0.2320	< 2e−16
		Season animal died (spring)	1.2988	0.2411	7.16e−08
		Season animal died (summer)	− 1.2927	0.3670	0.00043
		Distance to water	0.5016	0.1127	8.54e−06
		Carcass size	1.0577	0.1102	< 2e−16

The number of vertebrates actively scavenging at carcass sites (i.e., consuming species number) ranged from 0 to 8 species. Average consuming species number across all 13 sites was 4 ± 0.82 species. The best model for consuming species number included only habitat as a predictor (AIC_c_ = 63.4, adj. *D*^2^ = 42%), and parameter estimates indicated a higher diversity of consuming species at carcasses in grassland patches (*p* = 0.28) and a lower diversity at carcasses located in shrubland habitat (*p* = 0.4435), although neither parameter was considered significant. There were three additional models with a ΔAIC_c_ < 2. The only predictor included in the second-best model was seasonality (ΔAIC_c_ = 0.1, adj. *D*^2^ = 56%), the third-best model included elevation (ΔAIC_c_ = 0.3, adj. *D*^2^ = 29%), and the fourth-best model included elevation and carcass size (ΔAIC_c_ = 1.0, adj. *D*^2^ = 38%).

**Figure 2:**
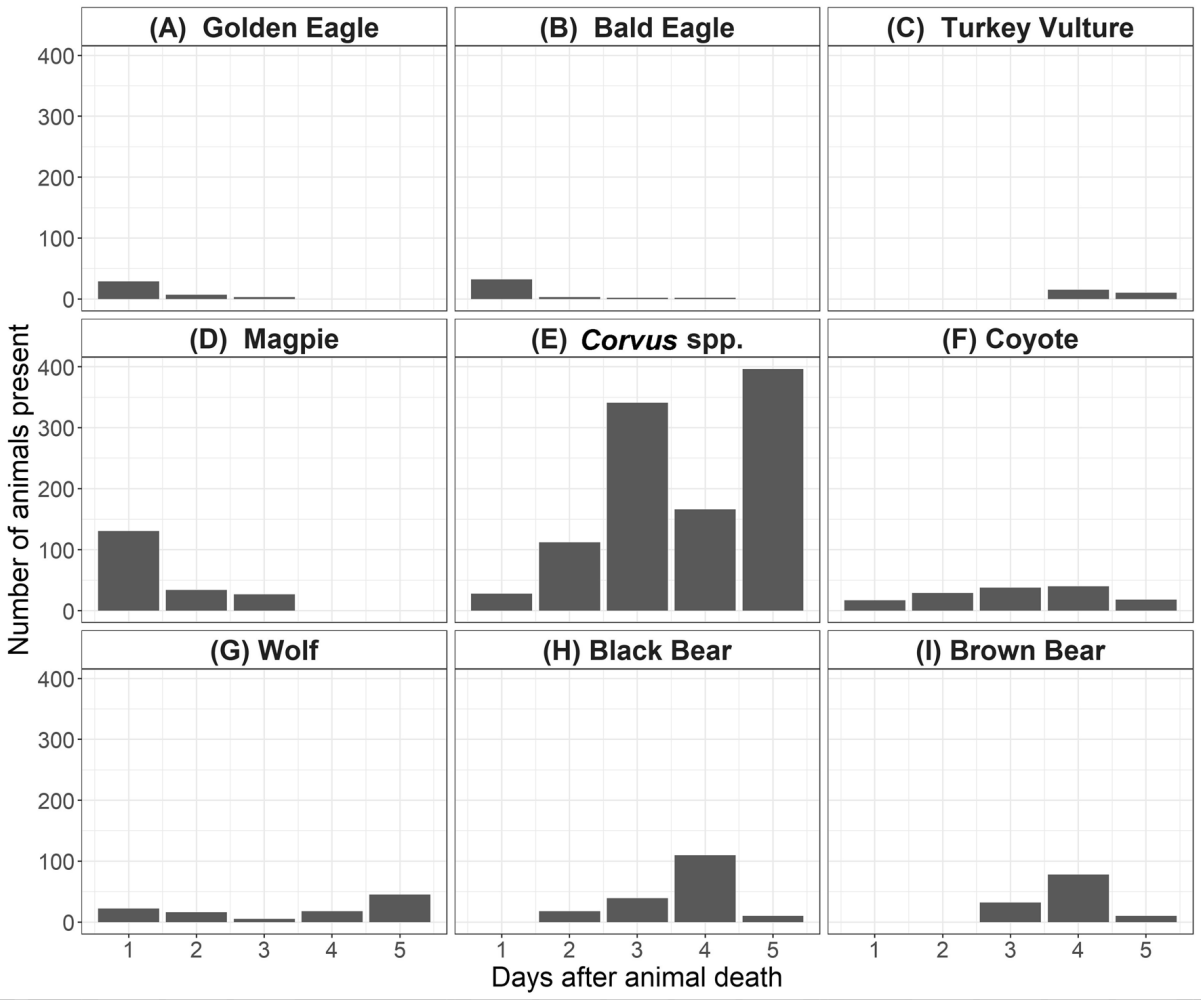
The numbers of different scavenger species captured by camera traps at carcass sites in southwestern Montana from 1 to 5 days after animal death. Magpies were present in the largest numbers initially, with *Corvus* spp. (ravens and crows) overall having the largest presence. Turkey vultures were absent in the initial days after death, a finding that is unique to this ecosystem. Coyotes and wolves were the first mammalian scavengers to arrive and usually had a shorter detection time than black and brown bears.

Camera traps were typically set up within 24 h of animal death and most carcasses (n = 9) were completely intact when cameras were activated, while four carcasses had already undergone some scavenging. For the nine intact carcasses, the mean detection time was 17.3 ± 5.2 h. Magpies, *Corvus* spp., eagles, wolves, and coyotes were the species that typically detected carcasses in the first 24 h after animal death (Fig. 2). Brown bears, black bears, and turkey vultures took 2–4 days to find carcasses. The best model for detection time included seasonality, the distance to the closest water body, the distance to the nearest road, and elevation as predictor variables (AIC_c_ = 107.5, adj. *D*^2^ = 87%). The parameter estimates indicated that scavengers detected carcasses faster in spring (*p* < 0.001), at carcass sites farther from water (estimate = -1.8846, p < 0.001), and in lower lying areas (*p* < 0.001). Conversely, detection time increased at carcass sites farther from major roads (*p* < 0.001) and in summer (*p* < 0.001).

The average amount of time it took for a carcass to be entirely consumed was 31.3 ± 9.8 days. From observation of camera trap photographs, if brown bears, black bears, or wolves were present at a carcass site, they dominated the consumption of carrion over smaller scavengers like coyotes or avian species. In the best-performing GLM of consumption time, the predictor variables included were seasonality, carcass size, and distance to the nearest water body (AIC_c_ = 76.5, adj. *D*^2^ = 98%). In contrast to detection time, the parameters for consumption time showed that consumption was slower in spring and faster in summer (spring *p* < 0.001; summer *p* < 0.001). Additionally, consumption time took longer at sites that were farther from water (*p* < 0.001) and at sites with larger carcasses (*p* < 0.001).

## Discussion

Here, we assessed the diversity and efficiency of the vertebrate scavenger guild in southwestern Montana, USA using camera traps at ungulate carcass sites. The compositions of scavenger guilds worldwide are affected by a host of environmental and anthropogenic factors, including NDVI, human footprint, habitat type, aquatic trophic conditions and season^32–36^. A predominance of facultative scavenging constituted most of the carcass removal in the study landscape. Turkey vultures are the only obligate scavenger species in Montana, and while vultures often dominate carrion consumption in other regions^37,38^, in this ecosystem most carcass scavenging was undertaken by bears, wolves, magpies, and *Corvus* spp. Perhaps the reason for this is because southwestern Montana is at the edge of turkey vultures’ northward range; in fact, populations are low enough that in some counties neighboring our study site, the Breeding Bird Survey has not recorded sightings of turkey vultures since 2000^39^. Yet previous work by Hill^40^ in South Carolina found that facultative mammalian scavengers were unable to functionally replace vultures, and similarly, studies in Spain^19^ and Kenya^23^ found that ungulate carcasses persist longer on the landscape in areas without vultures. While we cannot make direct comparisons here to vulture-exclusion studies, we did find that at all 10 sites where turkey vultures were absent, carcasses were either completely consumed (leaving only bones) or removed (dragged out of sight of the camera) within an average of 21.4 days. This efficiency of facultative scavenging could be related to the rich community of scavengers in this ecosystem, many of which are large carnivores.

Further, when turkey vultures were present at carcass sites, they did not arrive until 4 to 5 days after specimen death (Fig. 2). Instead, eagles, magpies, *Corvus* spp., coyotes, and wolves were the first species to detect and visit carcasses, usually on the same day the animal died or in the first day after animal death. When magpies and *Corvus* spp. are the first species to arrive at a carcass, they are often unable to open it without assistance from vultures or mammalian scavengers^41^. However, research on the exclusion of scavengers from carcasses found that vertebrate scavenging is not necessary for skin rupture^42^. Thus, quickly discovering carcasses may be advantageous to *Corvus* spp. and magpies, regardless of other scavengers present. As mentioned previously, longer detection times by vultures may be a function of a small population of individuals in this area, or the intensity of scavenging by mammalian vertebrates excluding them. Large carnivores are sometimes able to displace vultures at carcasses unless vultures are present in very high numbers^25^. We found here that when vultures were absent at carcasses, however, consuming species decreased from an average of 6.0 ± 1.2 species at sites *with* vultures to 3.5 ± 1.0 species for sites *without* vultures. This conflicts with previous findings about displacement, as well as findings in Africa that the total number of facultative mammalian scavengers and the time spent at carcasses by mammalian scavengers increased in the absence of vultures^23^.

Worldwide, 73% of vulture species are exhibiting population declines and 55% are considered endangered or critically endangered^43^. In this context, turkey vultures are an anomaly; they are one of the only obligate scavenger species currently undergoing population growth and range expansion. Kiff^44^ reports that since the 1950s, the range limits of both North American vulture species (turkey vultures and black vultures) have continued to undergo a steady expansion northward. Climate models suggest that an overall warming trend will continue to push the inhabitable territory for turkey vultures even further north^45^. Additionally, turkey vulture populations in the western U.S. increased by 3.8% per year from 1980–1996^46^. Though they provide crucial ecosystem services by removing carrion and mitigating disease risk, an increase in the vulture population in the montane ecosystem may have negative impacts on the availability of carrion to facultative scavengers. As obligate scavengers, turkey vultures are adapted to efficiently locate and consume carrion: they are typically the first species to arrive at a carcass^47^, can displace other species from feeding at carcass sites^48^, and can consume large portions of carrion^38^. We have shown here that carcasses are widely exploited by facultative scavengers, and previous research has found that scavenger species in this ecosystem are highly dependent on winter and spring carrion for overwinter survival and reproduction^31^. Thus, an increase in the turkey vulture population in this area and a subsequent increase in competition for carrion raises concern about the possible cascading effects for food webs in this ecosystem. Large carnivores have suffered widespread population declines^49^, and northwestern North America supports the highest number of large carnivore species on the continent (four species: black bears, brown bears, wolves, and pumas, *Puma concolor*). Increased resource competition over carrion would likely create new management and conservation challenges for protection of these carnivore species.

Additional anthropogenic stressors—not just warmer temperatures—could also create management and conservation challenges for scavengers. Conversion of forests to agro-grazing systems affects carrion resources by altering the abundance of carcasses and the habitats in which they occur, consequently impacting the availability and detectability of carcasses by scavengers. Arrondo et al.^18^ found that carcasses of domestic ungulates in open grazing areas were detected and consumed faster than those of wild ungulates in more heterogenous habitats. Here, we similarly found that grassland habitats had a higher number of consuming species than shrubland or forested areas, likely indicating increased competition in these open habitats. Additionally, mass mortality events due to environmental contamination, biotoxicity, and climate change from human perturbation are increasing^50,51^. During mass mortality events, ecosystems experiencing heavy loads of carrion (i.e. ≥ 360 kg/20 m^2^) are less efficient at recycling the additional available nutrients^50^, meaning facultative scavenger communities may have difficulty adapting to narrow, intense pulses of carrion resulting from anthropogenic disturbances.

We found that the diversity of scavenger species recorded visiting carcass sites and actively scavenging at carcass sites were both influenced by carcass size, with diversity increasing at larger carcasses in both cases. Previous studies have shown that carcass size can affect local scavenger guild structure^21,52^. For example, previous research in South Africa found that carcass size was a major factor determining the diversity of the associated scavenger assemblage, with the number of scavenger species and the speed of carrion removal both increasing as the size of the carrion increased^38^. Our findings were similar because smaller carcasses (3 elk carcasses, 1 yearling bison) had a mean visiting species value of 5 while larger carcasses (8 adult female bison, 1 adult male bison) had a mean visiting species value of 6.2. Likewise, smaller carcasses had a mean consuming species value of 3 and larger carcasses had a mean consuming species value of 5. Carcass size was also included as a predictor variable in models of consumption time, in which it took scavengers longer to consume larger carcasses than smaller ones.

Seasonality influenced all modeled response variables except the number of visiting species. However, the effect of season differed across response variables: a higher diversity of scavenging species was present at carcass sites when the animal died in summer, detection time was fastest in spring, and consumption time was fastest in summer (Table 2). Faster carrion consumption in summer could be an effort to prevent decay of carcasses at the hottest time of the year and accordingly, when decay rates peak^53–55^. Avoidance of rotting meat by scavengers has been previously documented; turkey vultures consume less carrion at older carcasses and in some cases avoid them altogether^38^, while pumas will regurgitate spoiled meat^56^. In summer, when temperatures are higher, vertebrate scavengers are also competing with microbes and arthropods for carrion^57,58^, which might promote faster carcass consumption along with faster decomposition rates. DeVault and Rhodes^59^ also found that ambient air temperature accounted for almost all variation in the number of carcasses removed by vertebrate scavengers. They argued that increased microbe activity at hotter temperatures allowed for stronger scavenger olfactory cues, which attracted more vertebrates to carcasses^59^. A possible explanation of faster detection time in spring could be the urgency with which animals are attempting to supplement their diets after undergoing a winter denning period or recovering from winter food scarcity. Predators face many difficulties when trying to acquire food in winter, as traveling through snow increases energy expenditures^60,61^ and recent snowfalls often dampen hunting success^62^.

Elevation was included as a predictor variable in several models for visiting species number, consuming species number, and detection time. The diversity of visiting and consuming species increased at higher elevations. A study from Yellowstone National Park found that brown bears were more likely to find and exploit carcasses at higher elevations due to their propensity to den at higher elevations^63^. This seemed true in our study: even though elevation varied from 1426 to 1900 m, brown bears were found scavenging at four out of the five sites located above 1830 m, and none of the sites below 1830 m. On the other hand, detection time was faster in lower-lying areas, probably due to the fact that carcasses decompose more quickly at lower elevations^64,65^. Faster decomposition and the olfactory signals that accompany it could lead to more rapid detection by scavenger species.

Our work examines the function of a diverse scavenger guild’s interactions with naturally occurring carrion instead of experimentally placed carcasses. Studies on scavenger dynamics at naturally occurring carcasses are rare^63,66^. Most scavenger studies use carcasses of animals that were purchased from commercial suppliers^40,47,67–69^, were provided by livestock farms^7^ or local breeders^59,70^, were killed by collisions with vehicles^56^, or were captured and euthanized in the field^64,69,71^. Studies of experimentally placed carcasses may differ from those using natural carcasses by artificially increasing the amount of biomass in the total carrion resource pool or by providing atypical visual and olfactory cues to scavengers for carrion detection. While using natural carcasses limited our sample size for this study, we argue that because of it, our results more accurately reflect the scavenging that occurs at spatiotemporally unpredictable^7^ carcass sites on this landscape.

In the study reported here, we found that scavenger guild diversity is influenced by traits about the carcass itself (particularly its size), the elevation and habitat of the carcass location, and the season. Most previous research in this ecosystem has focused on the reintroduction of wolves and the trophic cascade that followed^72–76^. Our study is novel in its examination of wolves as facultative scavengers rather than live prey hunters. Scavenging in this system is dominated by facultative scavengers, but the continued range expansion and population increase of turkey vultures may lead to increased competition between facultative and obligate scavengers over carrion. This could have cascading effects on food webs in the montane ecosystem and impact conservation of one of the few intact carnivore guilds in North America.

## Materials and methods

### Study site and data sampling

**Figure 3:**
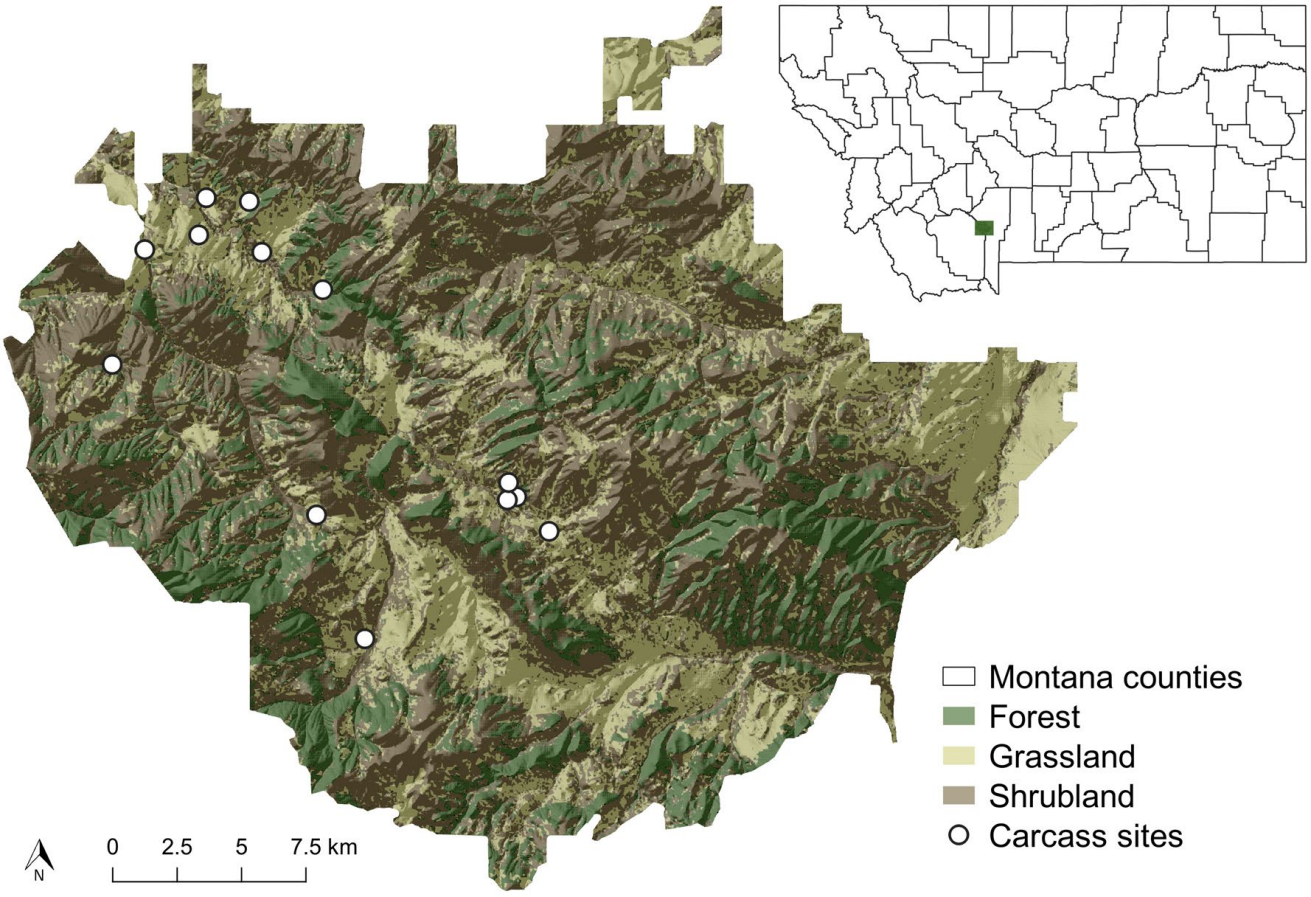
A map of the study area in southwestern Montana including the locations of carcass sites where camera traps were set, and the land cover classifications used in statistical analyses. Inset map indicates the position of the study area within the state of Montana. Land cover data were freely downloaded from the 2010 Montana Spatial Data Infrastructure (MSDI). land cover dataset (http://geoinfo.montanastatelibrary.org/data/msdi/landuse/) and simplified to three land cover categories. Elevation data were freely downloaded from a digital elevation model (DEM) developed using the United States Geological Survey (USGS) National Elevation Dataset (NED) with a 30-m resolution (http://ned.usgs.gov/). County data were freely downloaded from the www.gadm.org as administrative level 2 boundaries. Maps were produced in ArcGIS v 10.7 (www.esri.com; Redlands, CA, USA).

Camera trap monitoring was conducted on a ~ 300 km^2^ privately owned ranch in southwestern Montana from 2016 to 2019. The ranch is situated within the Madison range between the Madison and Gallatin Rivers and manages plains bison (*Bison bison bison*) as livestock (Fig. 3). While low fencing limits bison to the ranch itself, it does not restrict the movement of other wildlife. Camera trap stations were established at 13 carcass sites between 2016 and 2018 (five carcass sites in 2016, one in 2017, and seven in 2018). We opportunistically used a combination of bison (*n* = 10) and elk (*n* = 3) carcasses. Average nearest neighbor analysis^77^ (performed in ArcGIS v10.7) indicated that the carcass sites were significantly dispersed across the study area (nearest neighbor ratio: 1.285462; z-score: 1.969021; p-value: 0.048951). Cameras were placed approximately 10 m from the carcass at a height of 1–1.5 m, and programmed to take photos without delay, at a rate of 1 picture every second, once triggered by motion. Cameras were visited weekly to check battery levels and download photos from the external data cards; to control for human disturbance during camera trap maintenance, all traps were visited at the same frequency and for short periods of time (usually less than 10 min).

### Statistical analyses

We modeled the relationship among four different response variables to a set of predictor variables we hypothesized would influence scavenger diversity and scavenging efficiency. The response variables were considered: visiting species (the number of vertebrate scavenger species seen visiting the carcass at each site); consuming species (the number of vertebrate scavenger species observed consuming the carcass at each site); detection time (the number of hours elapsed until vertebrate scavengers started consuming the carcass at each site); and, consumption time (the number of hours elapsed from camera trap set up until the carcass was entirely consumed at each site). For four of the sites, there was already evidence of scavenging prior to camera trap set up. These were excluded from analyses of detection and consumption time, but still included in the visiting and consuming species analyses. Accordingly, the sample sizes for visiting species and consuming species GLMs were all 13 sites, while the sample sizes for consumption time and detection time were each 9 sites. For consuming species, a vertebrate scavenger was considered to have consumed the carcass if photographed tearing or pecking at the carcass, chewing, or if meat could be seen held in the mouth or beak. To account for the fact that camera traps were established in different years (2016–2018), visiting and consuming species counts were only conducted for the initial 4 months after camera trap set up.

Predictor variables included the habitat of the carcass (forest, grassland, or mixed shrubland), the carcass size (weight in kg), seasonality, distance to water, distance to roads, and elevation. Land cover classes were extracted from the Land Use/Land Cover data housed at the Montana Geographic Information Clearinghouse (http://geoinfo.msl.mt.gov/Home/msdi/land_use_land_cover, accessed Sep. 21, 2018). For elk and bison carcasses, body mass estimates were obtained from the literature^78–80^, accounting for sex and age. To test how the number of scavenger species and scavenging efficiency related to these predictor variables, we fitted separate generalized linear models (GLMs) for visiting species, consuming species, detection time, and consumption time. The predictor variables were tested for collinearity by calculating variance inflation factors (VIF) using the ‘car’ package in R 4.0.2^81,82^. Any variable with a VIF that exceeded 4 was excluded^83^, but the maximum VIF detected was 3.32. Predictor variables were also standardized by centering and scaling them around a mean of 0 with a standard deviation of 1. We used Poisson error distributions and log link functions. Model strengths’ were evaluated using Akaike’s information criterion for small sample sizes (AIC_c_)^84^. The quantities ΔAIC_c_ for each model were calculated as the differences in AIC_c_ between the model with the lowest AIC_c_ and each following model. We considered models with ΔAIC_c_ < 2 to be informationally indistinguishable in predicting the response variables^85^. As an additional measure of model fit, we also calculated an adjusted deviance

$$\mathrm{adj} {D}^{2}=\left(\frac{\mathrm{null\, deviance}-\mathrm{residual\, deviance}}{\mathrm{null \,deviance}}\right)\times 100$$

which represents the percent deviance explained by each model^86^.

Each model was tested for overdispersion using the AER package^87^. This package tests the null hypothesis of equidispersion in Poisson GLMs against the alternative of overdispersion or underdispersion. A significant *p*-value indicates the model is either over or under dispersed^88^. If the model was determined to be overdispersed, the Poisson was determined to be inappropriate, and a negative binomial model was used instead. However, significant deviance from Poisson dispersion was not detected in any model.

## Data Availability

Data and R code are available from https://doi.org/10.5281/zenodo.3901568.
